# The collection of birds from São Tomé and Príncipe at the Instituto de Investigação Científica Tropical of the University of Lisbon (Portugal)

**DOI:** 10.3897/zookeys.600.7899

**Published:** 2016-06-22

**Authors:** Miguel Monteiro, Luís Reino, Martim Melo, Pedro Beja, Cristiane Bastos-Silveira, Manuela Ramos, Diana Rodrigues, Isabel Queirós Neves, Susana Consciência, Rui Figueira

**Affiliations:** 1 CIBIO/InBIO-Centro de Investigação em Biodiversidade e Recursos Genéticos, Universidade do Porto, Vairão, Portugal; 2 CEABN/InBio, Centro de Ecologia Aplicada “Professor Baeta Neves”, Instituto Superior de Agronomia, Universidade de Lisboa, Tapada da Ajuda, 1349-017 Lisboa, Portugal; 3 CIBIO/InBIO-Centro de Investigação em Biodiversidade e Recursos Genéticos, Universidade de Évora, 7004-516 Évora, Portugal; 4 Percy FitzPatrick Institute of African Ornithology, University of Cape Town, Rondebosch 7701X, South Africas; 5 Museu Nacional de História Natural e da Ciência, Universidade de Lisboa, Rua da Escola Politécnica 56, 1250-102 Lisboa, Portugal; 6 CESAM-Centre for Environmental and Marine Studies, Universidade de Aveiro, 3810-193 Aveiro, Portugal; 7 MARE-FCUL, DOP/UAç - Departamento Oceanografia e Pescas, Univ. Açores, Rua Prof. Dr. Frederico Machado, 9901-862 Horta, Portugal; 8 Estrada de Mem Martins n251 1ºDto, 2725-391 Mem Martins, Sintra, Portugal

**Keywords:** Animalia, Aves, Chordata, Gulf of Guinea, Museum, Biodiversity databases, Species Occurrence data, Specimen

## Abstract

The former Instituto de Investigação Científica Tropical-IICT (Lisbon, Portugal), recently integrated into the University of Lisbon, gathers important natural history collections from Portuguese-speaking African countries. In this study, we describe the bird collection from the Democratic Republic of São Tomé and Príncipe, which was fully taxonomically checked and georeferenced. The IICT bird collection contains 5598 specimens, of which 559 are from São Tomé and Príncipe, representing 85 taxa, including 19 endemic species and 13 endemic subspecies of birds. The specimens were collected between 1946 and 1973, although 43% of the records are from 1954 and 45% are from 1970. The geographic distribution of samples covers the whole territory, with a higher number of records from São Tomé than from Príncipe. The districts with highest number of records are Pagué (equivalent to Príncipe Island), and Água Grande and Mé-Zochi on São Tomé. Despite the relatively low number of specimens per taxon, the importance of the collection is considerable due to the high number of endemic and threatened species represented. Furthermore, it adds valuable information to the GBIF network, especially for a country whose two islands are each an Endemic Bird Area and for which substantial gaps in ornithological knowledge remain.

## Introduction

The Democratic Republic of São Tomé and Príncipe is a country comprising two oceanic islands (São Tomé and Príncipe) and several islets located *ca.* 200 km from the coast of Gabon in the Gulf of Guinea, West Africa. From a bird dispersal perspective, the islands lie close enough to a biodiversity rich continental coast to make biological colonization likely, but sufficiently distant to allow successful colonizers to evolve in isolation from their mainland counterparts (Melo 2007). As a result, the endemism of bird species supported by each island is remarkable. In relation to their area, the number of endemic bird species is the highest globally and, although this is the second smallest country in Africa (*ca.* 1000 km^2^) it ranks in third place regarding the number of endemic birds (Stattersfield et al. 1998). Its forests have been considered the third most important in the world from a bird conservation perspective (Buchanan et al. 2011).

A total of 88 bird species are recorded for the islands (BirdLife International 2015), although that number increases to about 150 if vagrants are included (e.g., Christy 2001). There are 27 endemic species of which four are classified as Critically Endangered, one as Endangered and seven as Vulnerable (IUCN 2014, BirdLife International 2015; Suppl. material 1: Table S1). Twelve continental African bird species are represented by endemic subspecies (Table 2).

Despite the high numbers of endemic species, studies of the avifauna remained sporadic well into the 20th Century (Jones and Tye 2006). The importance of this unique avifauna was finally brought to the attention of the international conservation community during the 1980s (Collar and Stuart 1985, 1988). Based on the scarce literature available, the forests of São Tomé were ranked as the second most important of Africa and Madagascar from a bird conservation perspective (Collar and Stuart 1985) and seven species were tentatively classified as threatened following the criteria of the International Union for Conservation of Nature (IUCN) (Collar and Stuart 1988). Most importantly, these publications highlighted the worrying lack of up-to-date knowledge on the avifauna and the urgency in reversing this situation. New expeditions followed this call, rediscovering species not seen for over 60 years, including the São Tomé Grosbeak which was ‘lost to science’ for 101 years (Jones and Tye 1988, Atkinson et al. 1991, Sergeant et al. 1992). A steady number of research projects have continued since then (e.g., Christy and Clarke 1998, Melo and O’Ryan 2007, Melo and Fuchs 2008, Dallimer et al. 2009, 2010, Melo et al. 2010, 2011, de Lima et al. 2013, 2014).

Historical data from biological collections have played a central role in building-up our knowledge on the country´s avifauna (Amadon 1953, Frade 1958, 1959, Frade and Santos 1970, Jones and Tye 2006), especially when collections were made during periods without systematic ornithological sureys (Hromada et al. 2003, 2015, Leventis and Olmos 2009). Additionally, these collections are a source of valuable material for research including: i) taxonomic, phylogenetic and biogeographic studies; ii) diet studies from isotope analysis from feathers or nails; and iii) assessing changes in pollution by measuring heavy metal contents on feather samples collected at different point in the past.

This is the second of a series of data papers dedicated to the bird collection held by the Instituto de Investigação Científica Tropical of the University of Lisbon, following a previous one dedicated to birds from Angola (Monteiro et al. 2014). Here we provide a fully taxonomically revised and georeferenced dataset of the specimens from São Tomé and Príncipe, following the International Ornithological Congress taxonomic nomenclature (IOC World Bird List, v6.1) (Gill and Donsker 2016). The dataset is freely available online on the IICT IPT provider (http://maerua.iict.pt/ipt) and on the Global Biodiversity Information Facility (GBIF) data portal (http://www.gbif.org). It comprises 559 specimens from 107 different locations on both islands, collected between 1946 and 1973. Most specimens (491) were collected during two scientific expeditions that took place in 1954 and 1970, which were led by the collectors Fernando Frade (IICT) and René de Naurois (French naturalist that collaborated with IICT), respectively.

## General description

The bird collection of the Instituto de Investigação Científica Tropical (IICT), of the University of Lisbon holds a total of 5598 specimens, mainly from the Portuguese-speaking African countries: Mozambique, Angola, Guinea-Bissau, São Tomé and Príncipe, and Cape Verde. The dataset described here is the full subset from São Tomé and Príncipe, which contains 559 specimens that were taxonomically revised and georeferenced.

The collection comprises a high number of bird endemisms, including 19 out of 27 known endemic species of the country, together with an additional one shared with Annobón Island (Tables 1, Suppl. material 1: S1), and 13 of the 14 endemic subspecies described for the islands (Tables 2, Suppl. material 1: S2). The IICT collection holds 345 specimens from São Tomé Island, 213 from Príncipe Island and one lacking island information.

**Table 1.** Endemic species of the Republic of São Tomé and Príncipe represented in the IICT collection, including the number of specimens. The taxonomical nomenclature follows the IOC World Bird List v6.1 (Gill and Donsker 2016).

Common Name	Species	São Tomé (N)	Príncipe (N)	IUCN Red List (version 2014)
São Tomé Olive Pigeon	*Columba thomensis* Barboza du Bocage, 1888	11		Endangered
São Tomé Green Pigeon	*Treron sanctithomae* (Gmelin, JF, 1789)	7		Vulnerable
São Tomé Spinetail	*Zoonavena thomensis* (Hartert, 1900)	2	2	Least Concern
São Tomé Oriole	*Oriolus crassirostris* Hartlaub, 1857	6		Vulnerable
São Tomé Paradise Flycatcher	*Terpsiphone atrochalybeia* (Thomson, 1842)	14		Least Concern
São Tomé Prinia	*Prinia molleri* Barboza du Bocage, 1887	9		Least Concern
Dohrn's Thrush-Babbler	*Horizorhinus dohrni* (Hartlaub, 1866)		8	Least Concern
Príncipe Speirops	*Zosterops leucophaeus* (Hartlaub, 1857)		3	Near Threatened
Black-capped Speirops	*Zosterops lugubris* (Hartlaub, 1848)	15		Least Concern
São Tomé White-eye	*Zosterops feae* Salvadori, 1901	3		NA^1^
Príncipe Starling	*Lamprotornis ornatus* (Daudin, 1800)		13	Least Concern
São Tomé Thrush	*Turdus olivaceofuscus* Hartlaub, 1852	10		Near Threatened
Príncipe Sunbird	*Anabathmis hartlaubii* (Hartlaub, 1857)		11	Least Concern
Newton's Sunbird	*Anabathmis newtonii* (Barboza du Bocage, 1887)	11		Least Concern
Giant Sunbird	*Dreptes thomensis* (Barboza du Bocage, 1889)	5		Vulnerable
Príncipe Weaver	*Ploceus princeps* (Bonaparte, 1850)		14	Least Concern
Giant Weaver	*Ploceus grandis* (Gray, GR, 1844)	19		Least Concern
São Tomé Weaver	*Ploceus sanctithomae* (Hartlaub, 1848)	11		Least Concern
Príncipe Seedeater	*Crithagra rufobrunnea* (Gray, GR, 1862)	13	9	Least Concern

1 Treated by IUCN as a subspecies of *Zosterops ficedulinus*. Nominate subspecies occurs on Príncipe. Vulnerable status applies to the two populations.

**Table 2.** Endemic subspecies of African continental species present in São Tomé and Príncipe in IICT collection. The taxonomical nomenclature of the species follows the IOC Bird List v6.1.

Common Name	Species	Subspecies			
		São Tomé	N	Príncipe	N
Harlequin Quail	*Coturnix delegorguei*	*histrionica* Hartlaub, 1849	6		
Lemon Dove	*Columba larvata*	*simplex* (Hartlaub, 1849)	3	*principalis* (Hartlaub, 1866)	13
African Green Pigeon	*Treron calvus*			*virescens* Amadon, 1953	7
African Emerald Cuckoo	*Chrysococcyx cupreus*			*insularum* Moreau & Chapin, 1951	11
Western Barn Owl	*Tyto alba*	*thomensis* (Hartlaub, 1852)	2		
Little Swift	*Apus affinis*	*bannermani* Hartert, 1928	5	*bannermani* Hartert, 1928	9
Blue-breasted Kingfisher	*Halcyon malimbica*			*dryas* Hartlaub, 1854	9
Malachite Kingfisher	*Corythornis cristatus*	*thomensis* Salvadori, 1902	14	*nais* (Kaup, 1848)	5
Velvet-mantled Drongo	*Dicrurus modestus*			*modestus* Hartlaub, 1849	5
Chestnut-winged Starling	*Onychognathus fulgidus*	*fulgidus* Hartlaub, 1849	7		
Southern Masked Weaver	*Ploceus velatus*	*peixotoi* Frade & Naurois, 1964	14		

Each island is an independent Endemic Bird Area (Stattersfield et al. 1998), and their forests have been considered the third most important forests in the world for bird conservation, the other two being Hawaii tropical forests and Palau tropical moist forests (Buchanan et al. 2011). Although the original specimen labels lack full collecting information, it is possible to infer that the main collectors were Fernando Frade and René de Naurois. Fernando Frade visited the archipelago in 1954, and René de Naurois in 1970, precisely the years that aggregated 88% of the specimens (43% in 1954 and 45% in 1970).

## Records of special significance

This collection, although relatively small, provides a significant contribution to the ornithology of the islands as it was mainly put together from expeditions that took place in periods when otherwise no systematic ornithological surveys took place. In the 20^th^ century, the only other main collection efforts took place at the turn of the century and in 1928 (Jones and Tye 2006). The collection comprises specimens from 19 out of the 27 endemic bird species of the oceanic islands of the Gulf of Guinea: 10 single-island endemic species from São Tomé, 5 single-island endemic species from Príncipe, 4 endemic species present on both islands (Table 1) and one species endemic to Príncipe, São Tomé and Annobón islands. The IUCN Red List classifies four of these endemic species as Vulnerable and one as Endangered (Suppl. material 1: Table S1).

The collection also includes specimens from 13 subspecies from African continental species (Table 2). These include five specimens of *Dicrurus modestus* Hartlaub, 1849, which are of particularly interest as they may contribute to settle the long-standing debate on whether the population from Príncipe Island is a separate endemic species (*cf.* Jones and Tye 2006) – both by allowing specimens to be compared with mainland birds and by providing material for genetic analyses.

In addition, the collection is valuable in that it adds information to the existing data available through GBIF in terms of collecting dates and includes specimens of some endemic species for which there are few specimens in the collections worldwide, such as the São Tomé Green Pigeon (*Treron sanctithomae* (Gmelin, JF, 1789)), the São Tomé Olive Pigeon (*Columba thomensis* Barboza du Bocage, 1888) and the São Tomé subspecies of the Southern Masked Weaver (*Ploceus velatus peixotoi* Frade & Naurois, 1964). Finally, considering the size of the collection, it is surprising that it includes several specimens that represent the only known records for the islands: Pectoral Sandpiper (*Calidris melanotos* (Vieillot, 1819)), Great Spotted Cuckoo (*Clamator glandarius* (Linnaeus, 1758)), Red-footed Falcon (*Falco vespertinus* Linnaeus, 1766), Lesser Grey Shrike (*Lanius minor* Gmelin, JF, 1788) and Eurasian Golden Oriole (*Oriolus oriolus* (Linnaeus, 1758)).

## Taxonomic coverage

**Figure 1.**
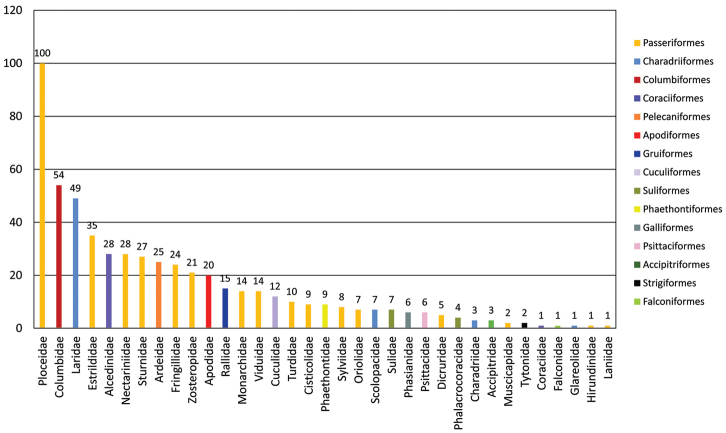
Total number of specimens per family. The legend lists the corresponding Orders, with assigned colors.

The IICT São Tomé and Príncipe bird collection comprises 15 orders and 35 families. The most represented orders are Passeriformes (54.7%), Charadriiformes (10.7%) and Columbiformes (9.7%). The families Ploceidae, Columbidae and Laridae are the ones with the highest number of records (100, 54 and 49, respectively) (Figure 1). The families Hirundinidae, Laniidae, Glareolidae, Falconidae, Coraciidae are each represented by a single record.

## Taxonomic ranks

**Kingdom**: Animalia

**Phylum**: Chordata

**Class**: Aves

**Order**: Accipitriformes, Apodiformes, Charadriiformes, Columbiformes, Coraciiformes, Cuculiformes, Falconiformes, Galliformes, Gruiformes, Passeriformes, Pelecaniformes, Phaethontiformes, Psittaciformes, Strigiformes, Suliformes

**Family**: Accipitridae, Alcedinidae, Apodidae, Ardeidae, Charadriidae, Cisticolidae, Columbidae, Coraciidae, Cuculidae, Dicruridae, Estrildidae, Falconidae, Fringillidae, Glareolidae, Hirundinidae, Laniidae, Laridae, Monarchidae, Muscicapidae, Nectariniidae, Oriolidae, Phaethontidae, Phalacrocoracidae, Phasianidae, Ploceidae, Psittacidae, Rallidae, Scolopacidae, Sturnidae, Sulidae, Sylviidae, Turdidae, Tytonidae, Viduidae, Zosteropidae

**Common names**: Birds

## Spatial and temporal coverage

**General spatial coverage**: São Tomé and Príncipe is a island country that consists of two archipelagos around two main islands, São Tomé and Príncipe, located in the equatorial Atlantic, in the Gulf of Guinea, at about 250 km west of the western equatorial coast of Africa. The islands are of volcanic origin, with maximum altitudes of 2024 m and 948 m for São Tomé and Príncipe, respectively.

**Figure 2.**
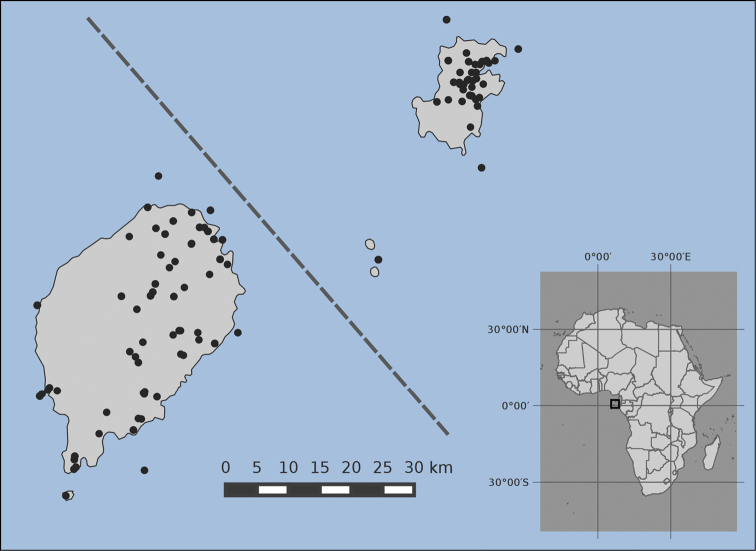
Distribution map of specimens occurrence throughout the territory of São Tomé and Príncipe. To facilitate graphic representation, distances between the two islands are not to scale (indicated by the dashed line).

The geographic range of the collection covers the whole territory of São Tomé and Príncipe (Figure 2). São Tomé Island has 345 records, while Príncipe Island has 213 records. The number of records per each of the seven districts of the country is as follows:

São Tomé Island - Água Grande (87), Mé-Zóchi (75), Caué (54), Lembá (51), Lobata (41) and Cantagalo (34). Príncipe Island - Pagué (213). For three records from São Tomé the district is unknown and for one record both district and island are unknown.

**Coordinates**: São Tomé (0°25'N and 0°01'S Latitude; 6°28'E and 6°45'E Longitude); Príncipe (1°32'N and 1°43'S Latitude; 7°20'E and 7°28'E Longitude).

**Figure 3.**
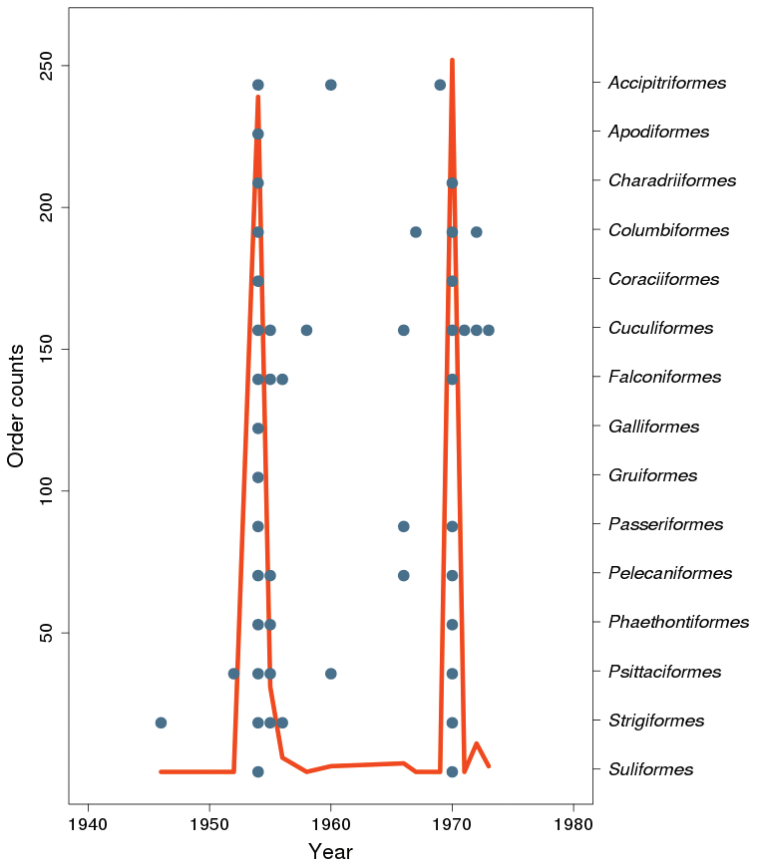
The sampling temporal profile of the collection´s specimens, showing the number of specimens per collection date. Blue dots represent sampling years for each Order.

**Temporal coverage**: The temporal range of the records lies between 1946 and 1973 (Figure 3). Most of the specimens (88%) were collected in two expeditions, which occurred in 1954 and 1970.

## Methods

**Method step description**: The collection of birds and mammals of IICT was catalogued for the first time under the project ARCA (2008-2010) using the software Specify Workbench and later imported to the collections’ database managed with the software Specify version 6 (Specify Software Project 2013). The catalogued information was transcribed but not revised or updated. Since 2012, the IICT bird collection has been taxonomically revised by the first author, with updates incorporated into the database. The cataloguing and georeferencing procedure followed Monteiro et al. (2014). Taxonomy followed the IOC Bird List (v6.1) (Gill and Donsker 2016), although the correspondence with taxonomy followed by BirdLife/IUCN is showed in Suppl. material 1: Tables S1 and S2. The information on the labels (collector, date of collecting, locality, descriptions of bill, eye and foot) was re-checked and the database corrected as necessary.

Since there were no geographic coordinates on labels or in associated record books, the georeferencing of specimen localities followed Chapman and Wieczorek (2006). The gazetteer Geolocate (Rios and Bart 2014) and Google Maps, were used to determine the coordinates and their uncertainty. The 1:25000 maps of São Tomé and Príncipe (IICT 1962, 1964) were used to search for coordinates not present on the gazetteers and, when possible, to fine-tune the positions. For four records there was no sufficient information to determine the geographic coordinates. The coordinates are given as decimal degrees using datum WGS 84.

**Study extent description**: The study covers both islands of the Democratic Republic of São Tomé and Príncipe. There are more samples for São Tomé (345) than for the smaller island of Príncipe (213). The best represented districts are Água Grande, Mé-Zochi (São Tomé Island) and Pagué (Príncipe Island).

**Sampling description**: Most of the records of the collection resulted from scientific visits or expeditions carried out between 1946–1973. There were two main collectors, Fernando Frade and René de Naurois, and two main dates 1954 and 1970. In 1954, the director of the Centre of Zoology of the Junta das Missões Geográficas e de Investigações do Ultramar (nowadays IICT-ULisboa), Fernando Frade, coordinated a three months scientific expedition to São Tomé and Príncipe (Missão Científica de São Tomé e Príncipe). In 1970, René de Naurois, visited the islands in one of his scientific trips (1963–1973) to collect and study São Tomé and Príncipe´s avifauna. Part of the scientific data gathered from the collected bird specimens were later published in the first book on the birds of the oceanic islands of the Gulf of Guinea (Naurois 1994).

**Quality control description**: For the development of the dataset the data from the labels of each specimen was revised by the first author because, initially, these had been transcribed as verbatim to the Specify 6 database. A taxonomic revision of the scientific names and a data checking were performed using IOC Bird List (version 6.1) followed by georeferencing according to the recommendations of Chapman and Wieczorek (2006), including the determination of uncertainty.

## Supplementary materials

Supplementary material 1 Species lists referred in Tables 1 and 2 showing correspondence between scientific and common names between IOC and BirdLife Internaction/IUCN nomenclatures Data type: species lists Explanation note: Nomenclatural differences between International Ornithological Council and BirdLife International/IUCN lists (2016) for endemic species and endemic subspecies of São Tomé and Príncipe. This dataset is made available under the Open Database License (http://opendatacommons.org/licenses/odbl/1.0/). The Open Database License (ODbL) is a license agreement intended to allow users to freely share, modify, and use this Dataset while maintaining this same freedom for others, provided that the original source and author(s) are credited.Miguel Monteiro, Luís Reino, Martim Melo, Pedro Beja, Cristiane Bastos-Silveira, Manuela Ramos, Diana Rodrigues, Isabel Queirós Neves, Susana Consciência, Rui Figueira
