# Supplementary material for: The collection of birds from São Tomé and Príncipe at the Instituto de Investigação Científica Tropical of the University of Lisbon (Portugal)
Source: Zookeys. 2016 Jun 22;(600):155–67. doi: 10.3897/zookeys.600.7899 (PMC4926686; doi:10.3897/zookeys.600.7899)
Supplement: Supplementary material 1 — Species lists referred in Tables 1 and 2 showing correspondence between scientific and common names between IOC and BirdLife Internaction/IUCN nomenclatures [file zookeys-600-155-s001.docx]

**TABLE S1: Endemic bird species of the islands of São Tomé (ST) and Príncipe (P) and their IUCN conservation status.**

Endemic status based in the taxonomy from the International Ornithological Council (IOC; Gill and Donsker 2016).

It differs slightly from the treatment of BirdLife International/IUCN list (2016). Nomenclatural differences between both lists are indicated. Both lists consider 27 endemic species for São Tomé and Príncipe islands, including one species shared with Annobón Island, 150 km south of São Tomé. Melo (2007) considers 28 endemics.

Differences between the lists are:

i) IOC treats *Zosterops ficedulinus* and *Z. feae* as two separate species (from Melo *et al.* 2011); whereas BirdLife/IUCN consider them two subspecies of *Z. ficedulinus*.

ii) BirdLife/IUCN consider the populations of kingfishers present on the two islands as two separate endemic species; IOC treats them as two subspecies of a mainland species (from Melo and Fuchs 2008; Table S2).

iii) Melo (2007) maintains the treatment of *Dicrurus modestus* as endemic to Príncipe as in Jones & Tye (2006) pending studies on the *Dicrurus adsimilis/modestus* complex. IOC considers the population from Príncipe as the nominate subspecies of a species with populations on the mainland. BirdLife/IUCN do not recognize *D. modestus* and lump it with *D. adsimilis*.

| **Scientific name** |  | **Common name** |  | **ISLAND** | **IUCN** |
| --- | --- | --- | --- | --- | --- |
| **IOC** | **BirdLife/IUCN** | **IOC** | **BirdLife/IUCN** |  |  |
| *Bostrychia bocagei* | = | Sao Tome Ibis | Dwarf Ibis | ST | CR |
| *Columba malherbii* | = | Island Bronze-naped Pigeon | Sao Tome Bronze-naped Pigeon | ST, P, A | NT |
| *Columba thomensis* | = | Sao Tome Olive Pigeon | = | ST | EN |
| *Treron sanctithomae* | = | Sao Tome Green Pigeon | = | ST | VU |
| *Otus hartlaubi* | = | Sao Tome Scops Owl | = | ST | VU |
| *Zoonavena thomensis* | = | Sao Tome Spinetail | = | ST, P | LC |
| *Lanius newtoni* | = | Sao Tome Fiscal | = | ST | CR |
| *Oriolus crassirostris* | = | Sao Tome Oriole | = | ST | VU |
| *Terpsiphone atrochalybeia* | = | Sao Tome Paradise Flycatcher | = | ST | LC |
| *Prinia molleri* | = | Sao Tome Prinia | = | ST | LC |
| *Horizorhinus dohrni* | = | Dohrn's Thrush-Babbler | Principe Thrush-babbler | P | LC |
| *Zosterops leucophaeus* | *Speirops leucophoeus* | Principe Speirops | = | P | NT |
| *Zosterops lugubris* | *Speirops lugubris* | Black-capped Speirops | = | ST | LC |
| *Zosterops ficedulinus* | *Zosterops ficedulinus* | Principe White-eye | Sao Tome White-eye | P | VU |
| *Zosterops feae* | *Zosterops ficedulinus* | Sao Tome White-eye | Sao Tome White-eye | ST | VU^1^ |
| *Lamprotornis ornatus* | = | Principe Starling | Principe Glossy Starling | P | LC |
| *Turdus olivaceofuscus* | = | Sao Tome Thrush | = | ST | NT |
| *Turdus xanthorhynchus* | = | Principe Thrush | = | P | CR |
| *Anabathmis hartlaubii* | *Nectarinia hartlaubii* | Principe Sunbird | = | P | LC |
| *Anabathmis newtonii* | *Nectarinia newtonii* | Newton’s Sunbird | = | ST | LC |
| *Dreptes thomensis* | *Nectarinia thomensis* | Giant Sunbird | = | ST | VU |
| *Ploceus princeps* | = | Principe Weaver | Principe Golden Weaver | P | LC |
| *Ploceus grandis* | = | Giant Weaver | = | ST | LC |
| *Ploceus sanctithomae* | = | Sao Tome Weaver | = | ST | LC |
| *Amaurocichla bocagii* | *Amaurocichla bocagei* | Sao Tome Shorttail | = | ST | VU |
| *Crithagra rufobrunnea* | *Serinus rufobrunneus* | Principe Seedeater | = | ST, P, B | LC |
| *Crithagra concolor* | *Neospiza concolor* | Sao Tome Grosbeak | = | ST | CR |

^1^: Species split not recognized by BirdLife/IUCN. VU status applies correctly to the rare population from Príncipe, but not to the common one from São Tomé.

A: Annobón Island; B: Boné de Jóquei islet, 2.5 km from Príncipe.

**TABLE S2: Subspecies of African mainland species that are endemic to the islands of São Tomé (ST) and Príncipe (P).**

*Dicrurus modestus* is often treated as an endemic species, the Príncipe Drongo (Jones and Tye 2006).

It is also likely that both *Onychognatus fulgidus* and *Columba larvata* constitute distinct species.

| **Scientific name** |  | **Common name** |  | **ISLAND** |
| --- | --- | --- | --- | --- |
| **IOC** | **BirdLife/IUCN** | **IOC** | **BirdLife/IUCN** |  |
| *Bostrychia olivacea rothschildi^1^* | = | Olive Ibis | = | P |
| *Coturnix delagorguei histrionica* | = | Harlequin Quail | = | ST |
| *Treron calva virescens* | = | African Green Pigeon | = | P |
| *Columba larvata simplex* | *Aplopelia larvata simplex* | Lemon Dove | = | ST |
| *Columba larvata principalis* | *Aplopelia larvata principalis* | Lemon Dove | = | P |
| *Chrysococcyx cupreus insularum* | = | Emerald Cuckoo | = | ST, P |
| *Tyto alba thomensis* | = | Barn Owl | = | ST |
| *Apus affinis bannermani* | = | Little Swift | = | ST, P |
| *Halcyon malimbica dryas* | = | Blue-breasted Kingfisher | = | P |
| *Corythornis cristata thomensis* | *Corythornis thomensis* | Malachite Kingfisher | Sao Tome Kingfisher | ST |
| *Corythornis cristata nais* | *Corythornis nais* | Malachite Kingfisher | Principe Kingfisher | P |
| *Dicrurus modestus modestus* | not split from *D. adsimilis* | Velvet-mantled Drongo | not split from Fork-tailed Drongo | P |
| *Onychognatus fulgidus fulgidus* | = | Chestnut-winged Starling | = | ST |
| *Ploceus velatus peixotoi* | *Ploceus vitelinus peixoti^2^* | Southern Masked Weaver | Vitelline Masked Weaver^2^ | ST |

^1^: Extinct. The species remains common on the mainland.

^2^: The BirdLife/IUCN name reflects the original but incorrect identification of the species.

**References Supplementary information**

BirdLife International (2016) Country profile: São Tomé e Príncipe. Available from: http://www.birdlife.org/datazone/country/sao-tome. Checked: 2016-04-08

Gill F, Donsker D, Eds. (2016) IOC World Bird List (v 6.1). doi:  10.14344/IOC.ML.6.1.

Jones P, Tye A (2006). The Birds of São Tomé & Príncipe with Annobón: Islands of the Gulf of Guinea. BOU Checklist Series 22. British Ornithologists’ Union & British Ornithologists’ Club, Oxford & Tring.

Melo M (2007) Bird Speciation in the Gulf of Guinea. PhD Thesis. University of Edinburgh, Edinburgh.

Melo M, Fuchs J (2008) Phylogenetic relationships of the Gulf of Guinea *Alcedo* kingfishers. *Ibis* 150: 633-639.

Melo M, Warren BH, Jones PJ (2011) Rapid parallel evolution of aberrant traits in the diversification of the Gulf of Guinea white-eyes (Aves, Zosteropidae). *Molecular Ecology* 20: 4953-4967.
